# Local Morphology Effects on the Photoluminescence Properties of Thin CsPbBr_3_ Nanocrystal Films

**DOI:** 10.3390/nano11061470

**Published:** 2021-06-01

**Authors:** Marco Anni, Arianna Cretí, Maria Luisa De Giorgi, Mauro Lomascolo

**Affiliations:** 1 Dipartimento di Matematica e Fisica “Ennio De Giorgi”, Università del Salento, Via per Arnesano, 73100 Lecce, Italy; marialuisa.degiorgi@unisalento.it; 2 IMM-CNR Institute for Microelectronic and Microsystems, Via per Monteroni, 73100 Lecce, Italy; arianna.creti@cnr.it (A.C.); mauro.lomascolo@cnr.it (M.L.)

**Keywords:** perovskite, nanocrystal, photoluminescence, time-resolved spectroscopy, confocal microscopy

## Abstract

Lead halide perovskites are emerging as extremely interesting active materials for a wide variety of optoelectronic and photonic devices. A deep understanding of their photophysics is thus fundamental in order to properly understand the origins of the materials active properties and to provide strategies for improving them. In this work, we exploit the local morphological variations in a drop-cast thin CsPbBr${}_{3}$ nanocrystal film to show that the aggregation of NCs has strong effects on the peak wavelengths of PL spectra, the linewidth, and the intensity of dependence on temperature. An analysis based on models that are frequently used in the literature led to completely different conclusions about the intrinsic NC emission properties extracted from spectra measured in points with different contribution of the PL from the aggregates. Our results demonstrate that extreme care has to be used in order to correctly correlate the spectral PL features with the intrinsic emission properties of lead halide perovskite NC films.

## 1. Introduction

Lead halide perovskite materials are currently receiving a huge amount of attention because of their promising combination of the active properties of standard semiconductors and the possibility of easy deposition in thin films from a solution.

Moreover, very fast and strong improvements in the performance of perovskite-based optoelectronic and photonic devices, including LEDs [1,2], solar cells [3,4,5,6,7], optically pumped active waveguides and lasers [8,9,10,11,12], and optical sensors [13,14], have been obtained in the last few years.

In particular, fully inorganic lead halide perovskite (CsPbX${}_{3}$) nanocrystals (NCs) are able to combine easy synthesis in a solution [15], high photoluminescence quantum yield (PLQY) [16,17], an ultra-wide color gamut [15,18], very high optical gain at room temperature [8,10], simple deposition onto thin films by using wet techniques [19], and improved stability with respect to organic–inorganic NCs [20]. Among these materials, CsPbBr${}_{3}$ NCs are particularly interesting for light-emitting devices in the visible spectrum due to their bright emissions in the green range, making them candidates for very promising semiconductors that are able to close the so-called “green-gap” that is normally found in semiconductors. This kind of NC was recently exploited for the demonstration of LEDs with an efficiency above 20% [21,22] and for low-threshold optically pumped lasers [23].

These strong application potentialities have stimulated basic physical investigations of the electronic properties of perovskite NCs in order to improve the understanding of the origin of their active properties and to provide possible further directions for improvement.

In this frame, a particularly powerful approach is the investigation of the temperature dependence of the photoluminescence (PL) spectra and of the PL relaxation dynamics that allows to investigate several features, such as the interplay between radiative and non-radiative relaxation processes, the origins of the emitting states (free carriers or excitons), and the coupling with phonons.

In particular, the analysis of the PL spectra shift (typically toward blue) and the increase in their linewidth as the temperature increases allows the quantitative investigation of exciton–phonon coupling [24,25,26,27,28,29]. In a similar way, the decrease in PL intensity as the temperature increases is typically ascribed to the thermal dissociation exciton, and the quantitative analysis of temperature-induced PL quenching allows the quantification of the exciton binding energy [26,27,28,30,31].

Despite the apparent simplicity of the determination of the fundamental quantities of CsPbBr${}_{3}$ NCs through the quantitative analysis of the temperature dependence of the PL spectra, a careful look at the values obtained in different experiments on nominally comparable samples discloses a complex scenario. In particular, even if it is usually claimed that the progressive increase in PL linewidth with the temperature is mainly related to the coupling of excitons with LO phonons, the reported values of the LO phonon energy are widely scattered in the range between 14.4 and 63.9 meV [26,27,28,29,30] and, most of the time, they are clearly inconsistent with the expected value of 20.5 meV [32]. Even more surprisingly, inconsistent values were also reported for the same sample in an analysis of the temperature-induced PL peak shift (55.6 meV) and the increase in linewidth (14.9 meV) [29]. In a similar way, the thermal activation of PL quenching is typically ascribed to the thermal dissociation of excitons, but the values obtained for the exciton binding energy are widely scattered in the range from 18.4 (even lower than the bulk CsPbBr${}_{3}$ value of 40 meV [15]) to 388.2 meV [26,27,28,29,30,33].

Finally, in puzzling contrast with the frequent evidence of temperature-induced PL quenching, several experiments on thin films of perovskite NCs [27,29,34,35,36,37] have curiously shown an increase PL intensity (anti-quenching) as the temperature increases. This effect was not observed in bulk polycristalline thin films or in single crystals of the same materials [27,35,38], and it has been tentatively ascribed to the thermal activation of the detrapping of excitons from surface dark states [35] or to the incomplete surface passivation of the ligands on the surfaces of the NCs [36].

In this work, by using measurements of temperature-dependent PL spectra and relaxation dynamics, we thoroughly investigated the emission properties of a CsPbBr${}_{3}$ NC film in order to improve the understanding of the origins of the different behaviors that are often shown by NC films with respect to bulk polycrystalline thin films or single crystals and to discuss the possible origins of the contrasting values in the literature for the LO phonon energy and for the exciton binding energy. Analogously to that which is typically done in similar experiments in the literature, drop casting was chosen as the deposition technique [24,26,28,30,34,35,36,39]. We demonstrated that the sample was made of a uniform NC film with a superimposed distribution of aggregates, which had a relative contribution to the total PL spectrum that was not uniform across the film; the local differences in NC aggregation determined strongly different emission properties, evidenced by variations in the PL peak wavelengths and in the temperature-induced PL peak wavelengths, linewidths, and intensity variations. In particular, we demonstrated that the PL intensity decreased as the temperature increased only in regions of the sample that showed a lower contribution of aggregation, while an interplay between temperature-induced PL intensity quenching and anti-quenching was observed in regions with emissions that were dominated by the aggregates.

Rather interestingly, the strongly different PL spectra temperature dependence were observed despite extremely similar temperature dependence of the PL relaxation dynamics.

The data analysis was performed in two steps, starting from a direct application of models that are typically used in the literature. In this case, we demonstrated that the best fit curves qualitatively reproduced the experimental data, but only with unphysical best fit values for the parameters. A more careful analysis allowed us to ascribe the inconsistencies to the temperature dependence of the aggregates contributions to the emission properties.

Overall, our results demonstrate that NC aggregation strongly affects the final emission properties of NC films and that any attempt to correlate the local emission properties of an NC film with the intrinsic NC properties must be made with extreme care and cannot avoid to investigate the film morphology.

## 2. Materials and Methods

### 2.1. Synthesis of the NCs and Film Preparation

**Figure 1:**
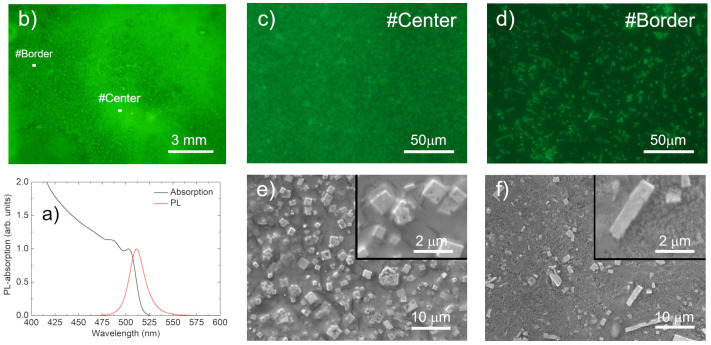
(**a**): Absorbance and PL spectra of the investigated NCs in solution. (**b**): Image of the sample from an optical microscope. The white rectangles show the typical morphologies of the regions in which the PL mapping was performed. (**c**): A 220 μm × 160 μm PL map at the #Center point. (**d**): A 220 μm × 160 μm PL map at the #Border point. (**e**): SEM image at the #Center point with 2000× and (inset) 6000× magnification. (**f**): e: SEM image at the #Border point with 2000× and (inset) 6000× magnification.

The nanocrystals were synthesized by following the procedure reported by A. Perulli et al. [40]; in solution, they showed a PL peak at about 512 nm and a clear excitonic absorption peak at about 503 nm (see Figure 1a).

The NC film was deposited by drop casting from a 10 mg/mL solution in toluene on a glass substrate, followed by natural drying in an air atmosphere.

### 2.2. PL and Time-Resolved PL Measurements

The PL and TR-PL measurements were performed as a function of the sample’s temperature in the range of 10–310 K in two positions on the film by exciting the sample with a solid-state pulsed laser (mod. PLP-10, Hamamatsu), which provided pulses at a wavelength of 400 nm for about 58 ps at a repetition rate of 1 MHz. The PL was dispersed with an iHR320 (focal length of 0.32 m) Horiba monochromator equipped with a Peltier cooled Hamamatsu photomultiplier (Head-on mod. R943-02), which operated in the single-photon-counting mode. The time-correlated single-photon-counting (TCSPC) technique was used to record the TR-PL with an Edinburg Instruments TCC900 TSCPC electronics card. The temporal resolution of the system was about 0.46 ns.

### 2.3. Investigation of the Morphology and PL Mapping

The sample morphology has been investigated at low magnification with a Dino-Lite AM4113T(R4) USB microscope.

Scanning electron microscopy (SEM) images were collected using a high-vacuum tungsten filament microscope (JEOL JSM-6480LV, dedicated software: SEM/JSM 5000) with a working bias of 5 kV. The elemental distribution was determined by using energy-dispersive X-ray (EDX) analyses (IXRF analyzer 500, dedicated software: EDS 2008) with a working bias of 20 kV.

PL mapping measurements were performed with a Nikon Eclipse C1 inverted fluorescence microscope with a 40× objective by exciting the samples in the 470–490 nm range with a mercury lamp and collecting the fluorescence in the 520–560 nm range.

## 3. Results

As a first step in our experiment, by using optical microscopy, we investigated the sample’s morphology (see Figure 1a). The image from the microscope clearly shows that deposition by drop casting leads to a thickness that progressively increases from the borders to the center, which is likely due to the faster evaporation of the solvent in the regions close to the borders.

In order to investigate the effects of the lack of uniformity on a smaller scale, we also measured the PL maps at two different points along the film surface by using fluorescence microscopy, which allowed us to observe (see Figure 1b,c) that the time needed for the evaporation of the solvent not only affected the sample thickness, but it also strongly affected the local morphology and emission properties of the film. In particular, the central region of the sample (#Center in the following) showed (see Figure 1b) a bright background emission with an almost uniform intensity, as well as a superimposed distribution of emitting microscopic structures. On the contrary, close to the film border (#Border in the following), we observed (see Figure 1c) a much weaker background emission intensity (which was consistent with the lower thickness), which led to a clearly increased difference in intensity between the background emission and the emissions of the micro-aggregates.

Further insight into the sample local morphology was obtained with SEM images, which allowed us to observe (see Figure 1e) that the brightly emitting micro-structures at the #Center point were almost cubic NC aggregates on top of a uniform film, with an edge length of a few microns. At the #Border point, a lower aggregate density was observed, on a less compact film (see Figure 1f).

The relative Cs:Pb:Br composition was determined with EDX measurements, obtaining similar values at both points (1:1.2:3.6 at the #Center point and 1:1.2:3.2 at the #Border point). These values were basically consistent with the nominal values (1:1:3).

The roles of the different relative contributions of the aggregates to the sample emission properties were investigated by using measurements of PL and time-resolved PL as a function of the sample temperature.

**Figure 2:**
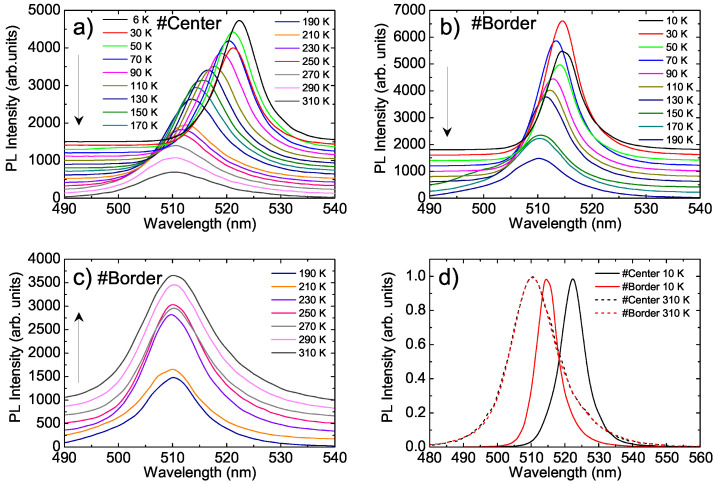
Temperature dependence of the PL spectra at the #Center point (**a**) and at the #Border point in the temperature ranges of 10–190 K (**b**) and 190–310 K (**c**). All of the spectra were vertically translated for clarity. The arrows show the direction of increasing temperature. (**d**): Comparison of the line shapes of the PL spectra at the two investigated points at low and room temperature. The spectra were normalized to 1 at the peak value.

The PL spectrum at T = 6 K at the #Center point (see Figure 2a) showed a single peak with a peak wavelength of about 522 nm and a full width at half maximum (FWHM) of about 8.1 nm (corresponding to 37 meV). As the temperature increased, the PL peak wavelength underwent a progressive blue-shift from about 522 nm to about 510 nm, the FWHM increased, and the PL intensity progressively decreased for temperatures above 110 K; these variations were qualitatively similar to those found in many previous reports on similar materials [24,25,26].

When the same kinds of measurements were performed at the #Border point, clearly different PL spectra were observed. In particular, the spectrum (see Figure 2b) at T = 6 K was peaked at 514 nm, thus blue-shifted of about 8 nm with respect to the one at the #Center point. In addition, it was narrower with a FWHM decreased to 7.2 nm (34 meV). The observed dependence of the PL intensity on temperature was also very different from that at the #Center point with an almost stable value up to 130 K, followed by a quenching up to 190 K (see Figure 2b) and a clear recovery of intensity up to room temperature (see Figure 2c), leading to an overall increase in PL intensity of about 70% from 10 K to room temperature, while a quenching of about 60% was observed at the #Center point.

Also in the #Border point we observe a PL peak wavelength blue-shift and a FWHM increase as the temperature increases and both values progressively approach the corresponding values of the #Center point up to room temperature, at which identical PL line-shapes are observed in the two points (see Figure 2d).

**Figure 3:**
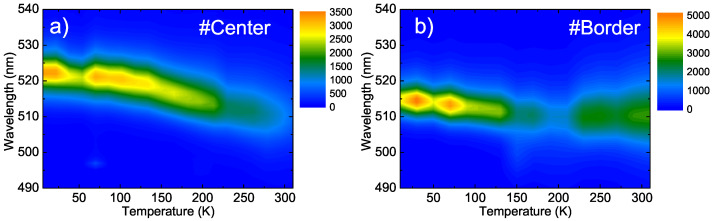
Two-dimensional map showing the temperature dependence of the PL at the #Center point (**a**) and #Border point (**b**).

The different PL line-shapes and temperature dependence can be more easily visualized through the comparison of 2D spectral maps (see Figure 3).

**Figure 4:**
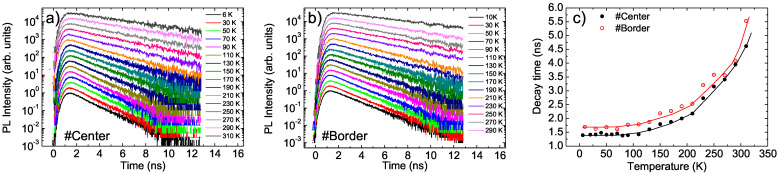
Temperature dependence of the PL relaxation dynamics at the #Center (**a**) and #Border (**b**) points. The data were normalized to the peak values and vertically scaled for clarity. (**c**): Temperature dependence of the PL decay time at the two investigated points (the lines are for visual guidance).

In order to further investigate the differences in the emission properties of the two points, we also performed time-resolved PL measurements with the aim of probing the exciton relaxation dynamics. The PL relaxation dynamics at the #Center point (see Figure 4a) showed a mono-exponential decay in the whole temperature range with a temperature-independent lifetime of about 1.4 ns up to 110 K, and then a progressive increase in lifetime up to 4.6 ns at room temperature (see Figure 4c) [24,25,27,33].

Rather interestingly, the PL relaxation dynamics at the #Border point were almost identical to those at the #Center point (see Figure 4b), despite the clearly different temperature dependence of the PL spectra, showing a mono-exponential relaxation over the whole temperature range, with decay times that were slightly higher than at the #Center point, but with a very similar temperature dependence (see Figure 4c).

## 4. Discussion

Our results clearly show that the PL spectra and their dependence on temperature are strongly affected by local differences in the relative importance of the emissions of the NC film and the NC aggregates. This aspect is particularly relevant because the experiments in the literature that exploited the temperature dependence of the PL intensity and the relaxation time to understand the intrinsic photo-physics of lead halide perovskite materials typically did not pay any particular attention to the local morphologies of the points that were excited.

In order to investigate the possible consequences of the direct correlation of the temperature dependence of the PL and TR-PL with the basic photo-physics, neglecting the local morphology of the excited point, in Section 4.1 we will separately analyze the results obtained at the two points, simulating a situation in which only one position is investigated, as is typically done in the literature.

We will show that this easy approach will lead to the possibility of individually finding an explanation for the observed results, but for two inconsistent pictures of the CsPbBr${}_{3}$ NCs photo-physics. In Section 4.2, we will then examine the results in greater detail in order to gain a consistent understanding of our results.

### 4.1. Conventional Analysis of the Two Points

First, we will investigate the emission properties of the #Center point by starting from the temperature dependence of the PL intensity.

**Figure 5:**
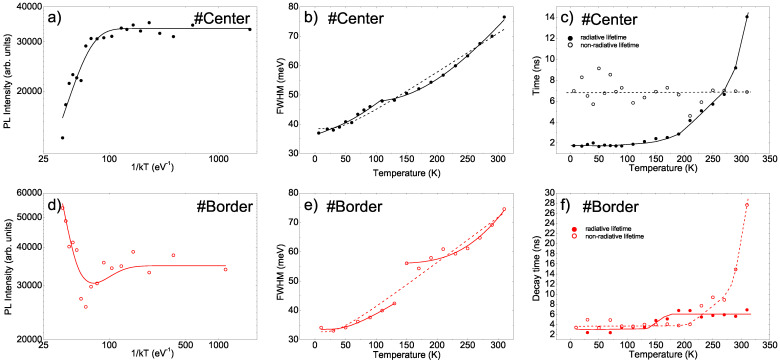
(**a**): Temperature dependence of the integrated PL intensity at the #Center point (dots). The line is the best fit curve obtained with Equation (1). (**b**): Temperature dependence of the FWHM of the PL spectra at the #Center point (dots). The dashed line is the best fit curve obtained with Equation (2) over the entire temperature range (Section 4.1), while the two continuous lines are the best fit curves for temperatures reaching up to 110 K and above 110 K (Section 4.2). (**c**): Temperature dependence of the radiative and non-radiative lifetimes at the #Center point obtained from Equations (3) and (4). The curves are for visual guidance. (**d**): Temperature dependence of the integrated PL intensity at the #Border point (dots). The line is the best fit curve obtained with Equation (5). (**e**): Temperature dependence of the FWHM of the PL spectra at the #Border point (dots). The dashed line is the best fit curve obtained with Equation (2) over the entire temperature range (Section 4.1), while the two continuous lines are the best fit curves for temperatures reaching up to 130 K and above 130 K (Section 4.2). (**f**): Temperature dependence of the radiative and non-radiative lifetimes at the #Border point obtained from Equations (3) and (4). The curves are for visual guidance.

The evidence of a stable intensity at low temperatures followed by thermally induced quenching (see Figure 5 a) can be reproduced by assuming the coexistence of a temperature-independent radiative lifetime $\tau_{r}$ and a thermally activated non-radiative lifetime $\tau_{nr}=\tau_{0}exp\left(\Delta E/k_{B}T\right)$, where $\tau_{0}$ is a constant, ΔE is the activation energy, and $k_{B}$ is the Boltzmann constant. The temperature dependence of the PL intensity is thus given by the following Arrhenius equation [38]:

(1) $$I(T)=\frac{I_{0}}{1+\frac{\tau_{r}}{\tau_{0}}e^{-\frac{\Delta E}{k_{B}T}}}$$

A good agreement between the experimental value and the best fit curve was obtained for ΔE=(67±10) meV (see Figure 5a), that is within the range of the typical thermal quenching activation energy in thin CsPbBr${}_{3}$ films, usually ascribed to the exciton binding energy [26,27,28,30,31].

Concerning the temperature dependence of the FWHM, the progressive broadening that was observed as the temperature increased (see Figure 5b) is typically related to the coexistence of an inhomogeneous temperature-independent broadening and a temperature-dependent contribution from exciton–phonon coupling, leading to the following equation [26,27,28,31,33,35]:

(2) $$FWHM(T)=\Gamma_{in}+AT+\Gamma_{LO}{\left(e^{E_{LO}/k_{B}T}-1\right)}^{-1}$$

where $\Gamma_{in}$ is the contribution of the inhomogeneous broadening, A and $\Gamma_{LO}$ are the coupling constants with acoustic and LO phonons, respectively, $E_{LO}$ is the LO phonon energy, and $k_{B}$ is the Boltzmann constant.

The best agreement between the experimental data and the fit curve (see Figure 5b) was obtained for the values of the best fit parameters that are shown in Table 1 (first row). In particular, we observed that the best fit was obtained by setting A = 0, allowing us to conclude that the increase in broadening was due to coupling with LO phonons with a phonon energy of 11±4 meV.

Finally, by exploiting the knowledge of both the PL intensity and the PL decay time, we also determined the radiative and non-radiative decay times as a function of the temperature by starting from $I_{PL}\left(T\right)=Ag_{0}\eta \left(T\right)$, where $I_{PL}\left(T\right)$ is the PL intensity at temperature T, η(T) is the PL quantum yield (PLQY) at temperature T, $g_{0}$ is the number of photons absorbed, and A is a constant scale factor. Moreover, we have $\eta \left(T\right)=\tau \left(T\right)/\tau_{rad}\left(T\right)$, where τ(T) and $\tau_{rad}\left(T\right)$ are the total and the radiative lifetimes at temperature T, respectively. Assuming a known value of the PLQY at 10 K ($\eta \left(10K\right)$) and by calculating the ratio between the PL intensities at low temperatures and the those at a generic temperature *T*, we have:

(3) $$\tau_{rad}(T)=\frac{\tau \left(T\right)}{\eta \left(10K\right)}\frac{I_{PL}\left(10K\right)}{I_{PL}\left(T\right)}$$

(4) $$\tau_{nrad}(T)={\left(\frac{1}{\tau \left(T\right)}-\frac{1}{\tau_{rad}\left(T\right)}\right)}^{-1}$$

We estimated η(10K)≈80% by considering that the typical PLQY of CsPbBr${}_{3}$ NC films at room temperature is about 30% [41] and that the increase in PL intensity from room temperature to 10 K at the #Center point is of about 2.5 times [42].

By using the experimental values of the PL intensities $I_{PL}\left(T\right)$ and of the decay times τ(T), we then obtained the values of $\tau_{rad}\left(T\right)$ and $\tau_{nrad}\left(T\right)$ at each temperature (see Figure 5c), suggesting that the variation in the PL intensity resulted from the interplay between an almost temperature-independent non-radiative lifetime of about 7 ns and a strongly increasing radiative lifetime, from 1.7 ns at 6 K to 14 ns at room temperature. This result would allow us to ascribe the temperature-dependent PL quenching to a progressive transition toward emitting sites with higher radiative lifetimes, rather than to a temperature-induced decrease in the non-radiative lifetime, as is usually assumed in the literature (and in all analyses based on Arrhenius plots).

The same analysis was repeated for the data obtained at the #Border point. In this case, the temperature dependence of the PL intensity (see Figure 5d) showed quenching, followed by an increase in intensity that could be modeled by assuming the presence of a non-radiative thermally activated process that determined the quenching, as well as a thermally induced process that enhanced the PL (similarly to a detrapping). In this case, the Arrhenius equation became [38]:

(5) $$I(T)=\frac{I_{0}}{1+\frac{\tau_{r}}{\tau_{0}}e^{-\frac{\Delta E}{k_{B}T}}-\frac{\tau_{r}}{\tau_{1}}e^{-\frac{\Delta E_{2}}{k_{B}T}}}$$

in which the latter term was responsible for the thermally induced PL enhancement, with an activation energy $\Delta E_{2}$.

The best fit (see Figure 5d) was obtained for the activation energy of the PL quenching process of 40 ± 10 meV and the activation energy of the PL enhancement process of 52 ± 10 meV.

The temperature dependence of the FWHM was reproduced (see Figure 5e) by the best fit with Equation (2), with the best fit parameters reported in Table 1. Also in this case the best fit is obtained with A = 0, evidencing the lack of significant role of acoustic phonons, and a best fit LO phonon energy of 9 ± 6 meV was found.

Finally, we determined the temperature dependence of the radiative and non-radiative decay times. We obtained (see Figure 5e) completely different behaviors with respect to the #Center point. In particular, we found a temperature-independent non-radiative decay time of about 4 ns up to 210 K, followed by a strong increase to 28 ns at room temperature. The radiative decay time basically remained constant at around 3 ns up to 130 K; then, it increased to about 6.5 ns between 130 and 190 K, and stayed constant again up to room temperature. These results would now allow us to ascribe the quenching of PL intensity to an increase in the radiative lifetime and the strong anti-quenching to the suppression of a non-radiative decay process.

Overall, the two pictures of the basic photo-physics that we could make on the basis of the two individual sets of measurements are clearly inconsistent. In particular, a correct analysis of the LO phonon energy obtained from the broadening temperature dependence should honestly lead to evidence that the best fit values obtained at the #Center point and the #Border point (11±4 meV and 9±6, respectively) are compatible each other, but they are clearly not compatible with the value of 20.5 meV expected for CsPbBr${}_{3}$ [32]. On the other hand, the huge error bar for the value at the #Border point should raise doubts about the real applicability of Equation (2) for the correct reproduction of the origins of the linewidth of the PL spectra.

Another element of inconsistency comes from the discussion of the radiative or non-radiative origins of the variations in PL intensity obtained through the separation of the two lifetimes based on a rather standard assumption of the correlation between the PL intensity and the total decay time. In particular, the radiative lifetime is an intrinsic property of the material and, thus, cannot be affected by the local morphology (which could instead likely affect the role of non-radiative defects). On the contrary, the data of the two points indicate a completely different temperature dependence of the radiative lifetime, which does not make any sense.

Overall, our results clearly demonstrate that extreme care in the analysis of the data must be taken in order to correctly understand the basic photo-physics of lead halide perovskite NC films through temperature-dependent PL and TR-PL measurements.

### 4.2. Correct Analysis

In this sub-section, we will repeat the data analysis with the necessary care in order to remove the inconsistencies among the conclusions obtained above and to develop a complete understanding of the emission properties of the sample, starting from the temperature dependence of the PL linewidth, which, according to the previous analysis, cannot simply be ascribed to coupling with LO phonons of an inhomogeneously broadened system.

A careful look at the values of the temperature dependence of the FWHM allows to observe, at the #Center point (see Figure 5b), a clear kink at 110 K, followed by an evident variation in the functional dependence of the FWHM on the temperature; in addition, at the #Border point (see Figure 5e), there was a clear discontinuity between 130 and 150 K. This behavior is evidence of the presence of two different regimes, and it strongly suggests that two different kinds of emitters with different line-width temperature dependence contribute to the sample PL, with the first dominating at low temperatures and the second at high temperatures.

The use of Equation (2) to reproduce whole FWHM temperature dependence thus qualitatively reproduced the data (as basically both the data and the best fit curves were increasing functions with some curvature), but provided unphysical best fit values for the parameters because our system is not made by an inhomogeneously broadened system with a single kind of emitter interacting with the phonons.

A correct analysis must instead consider the presence of two different temperature regimes of the increase in the FWHM, thus performing two separate best fit analyses with Equation (2) in the two temperature ranges. Concerning the #Center point, we observed (see Figure 5b and Table 1) that the broadening up to 110 K could be fully ascribed to coupling with LO phonons, with a best fit LO phonon energy of 20 ± 3 meV, which is now fully consistent with the value expected for CsPbBr${}_{3}$. Above 110 K, the PL broadening was qualitatively similar, though weaker, and could be correctly reproduced with a higher value of the characteristic energy (57 ± 5 meV), which is instead not compatible with the LO phonon energy of CsPbBr${}_{3}$. A similar result was obtained at the #Border point (see Figure 5e), which showed a good best fit of the broadening up to 130 K with a negligible role of acoustic phonons, and with a best fit LO phonon energy of 15 ± 13 meV, which, despite being affected by a larger error bar, is again compatible with the expected value for the LO phonon energy of CsPbBr${}_{3}$. In this case, the broadening at higher temperatures is characterized by a much higher characteristic energy (121 ± 18 meV), which cannot be ascribed to the LO phonon energy of CsPbBr${}_{3}$.

Overall, these results allow us to ascribe the observed temperature-induced PL broadening to the coupling of LO phonons only in the low temperature range, showing that only in this range the sample emission can be ascribed to the intrinsic emissions of the NCs.

The knowledge of the local morphology of the two investigated points also allowed us to understand the origins of the different PL peak wavelengths at the low temperature, that can be ascribed to the different packing of the NCs in the uniform part of the film. In particular, the longer evaporation time at the #Center point determine a closer NC packing, allowing the migration of energy within the size distribution of the NC and leading to emission dominated by larger NCs that emitted at higher wavelengths [10,25]. When the temperature increased up to 110 and 150 K for the #Center and the #Border points, respectively, the PL broadening showed a kink, proving that other states beyond excitons in the NCs started to contribute to the emissions, and the PL intensity started to decrease. This result can be ascribed to the thermal activation of exciton transfer from the uniform film to the aggregates, thus changing the functional dependence of the broadening on the temperature, as a second family of states started to contribute to the emissions and causing a PL quenching, consistent with a lower PLQY of the aggregates with respect to the uniform film.

This attribution also led to the reconsideration of the meaning of the PL quenching activation energy, which is likely not related to thermal quenching of excitons, but instead to the thermal activation of exciton migration towards the aggregates, thus giving a sense to the wide range of PL quenching activation energy reported for CsPbBr${}_{3}$ NC films in the literature [26,27,28,29,30,33].

Concerning the PL anti-quenching, we observed its presence only at the #Border point, that was characterized by a higher relative aggregate/film PL intensity, suggesting that the enhancement of the PL intensity due to the detrapping process was likely related to the effects surface defects as a result of incomplete passivation by the ligand, as postulated in previous literature [35,36,43], but this mainly involves aggregated NCs, but not individual isolated NCs.

A last aspect that must be discussed is the inconsistency between the temperature dependence of the radiative and non-radiative decay times at the two points. Excluding the possibility that the same material can have two completely different temperature dependence of the radiative lifetime, which is an intrinsic property that is not affected by the different local morphology, our result clearly demonstrates that the assumed correlation between the PL intensity and the total decay time, which is standard for semiconductors, does not hold for perovskite NCs. This is also suggested by the basically identical PL relaxation dynamics at the two points, despite the completely different temperature dependence of the PL spectra (intensity, position, and FWHM). The similarities in the PL relaxation dynamics suggest that the TR-PL allows to probe the same relaxation processes at the two points, that is likely related to the exciton relaxation in the uniform film. On the contrary, the line shape and intensity of the PL were clearly affected by the contributions of the aggregate emissions, whose relaxation was evidently not visible in the TR-PL measurements (likely because they had a much longer lifetime). This situation invalidated the assumed correlation between the PL intensity and decay time, thus leading to a nice evolution in temperature of the radiative and non-radiative decay times, that however does not have a real physical meaning. Our conclusions are fully consistent with the recent results concerning the lack of correlation between the relaxation dynamics of the PLQY and PL [43] in perovskite NC films, which was ascribed to the contributions of shallow surface-related defects to the emission and relaxation dynamics but, in addition, suggest that the surface-related exciton trapping and detrapping processes are mainly present in aggregated NCs than at the individual NC scale.

**Figure 6:**
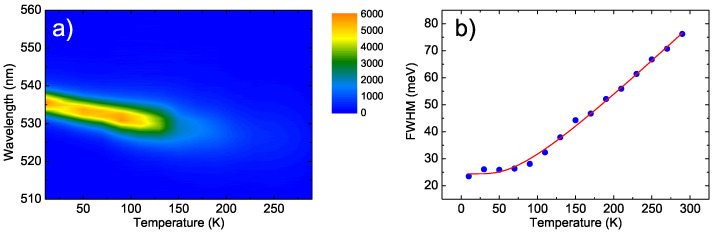
(**a**): Two-dimensional map of the temperature dependence of the PL of the bulkCsPbBr${}_{3}$ polycrystalline thin film. (**b**): Temperature dependence of the FWHM of the PL spectra of the same sample (dots) and the best fit with Equation (2).

In order to indirectly confirm the role of NC aggregates in determining the emission properties of the film, we also investigated the temperature dependence of the PL of a uniform bulk CsPbBr${}_{3}$ polycrystalline film, which was deposited by spin coating a mixture of CsBr and PbBr${}_{2}$ in solution with a 2:1 CsBr:PbBr${}_{2}$ molar ratio [44]. The 2D map of the temperature dependence of the PL spectra (see Figure 6a) clearly shows a progressive blue-shift and a decrease in the intensity of the spectra, which is fully consistent with the results previously reported for a nominally identical film [38]. The temperature dependence of the FWHM (see Figure 6b) showed a progressive increase that could be excellently reproduced by the best fit curve with Equation (2) without contributions from acoustic phonons and with a best fit LO phonon energy of 19 ± 2 meV, which is fully consistent with the expected value (see Table 1).

## 5. Conclusions

In conclusion, by using PL, TR-PL, optical, fluorescence, and electron microscopy, we investigated the effects of NC aggregation on the emission properties of thin CsPbBr${}_{3}$ NC films.

We demonstrated that the line shape and intensity of the sample emissions were determined by the interplay between intrinsic NC emission and the emission of the aggregates, whose relative contributions were very different across the film surface.

Overall, our experiment clearly showed that the direct application of models that are typically used for the description of the temperature dependence of the PL of standard semiconductors to lead halide perovskite NC films exposes researchers to risks of incorrect conclusions concerning the origins of the spectral properties observed when NC aggregation is present. On the other hand, the very different behaviors of our sample in the two different positions clearly showed that the possible non-uniformity of local morphology, for example induced through deposition by drop casting, can strongly affect the photo-physics of thin perovskite films, making crucial the control of the uniformity of the morphology before drawing conclusions about the intrinsic properties of the material based on an analysis of the PL spectra and relaxation dynamics.

## Figures and Tables

**Table 1:** Best fit values of the parameters extracted from the analysis of the temperature dependence of the FWHM of the two investigated points of the NC film and the bulk polycrystalline thin film. LT and HT are relative to the fit and represent low temperature and high temperature, respectively.

#Point	$\Gamma_{\mathrm{in}}$	A	$\Gamma_{\mathrm{LO}}$	E${}_{\mathrm{LO}}$
	**(meV)**	**(meV/K)**	**(meV)**	**(meV)**
#Center	38.5 ± 1.0	-	18 ± 7	11 ± 4
#Center-LT	36 ± 7	-	25 ± 13	20 ± 3
#Center-HT	47.5 ± 0.6	-	214 ± 40	57 ± 5
#Border	33 ±2	-	17 ± 13	9 ± 6
#Border-LT	33.5 ± 0.6	-	24.6 ± 10	15 ± 3
#Border-HT	56.0 ± 1.1	-	1.7 ± 1.2	121 ± 18
Film	24.4 ± 0.7	-	60 ± 8	19 ± 2

## Data Availability

The data is included in the main text.
